# Climate Change Forces New Ecological States in Tropical Andean Lakes

**DOI:** 10.1371/journal.pone.0115338

**Published:** 2015-02-03

**Authors:** Neal Michelutti, Alexander P. Wolfe, Colin A. Cooke, William O. Hobbs, Mathias Vuille, John P. Smol

**Affiliations:** 1 Paleoecological Environmental Assessment and Research Lab (PEARL), Department of Biology, Queen’s University, Kingston, Ontario, K7L 3N6, Canada; 2 Department of Biological Sciences, University of Alberta, Edmonton, AB T6G 2E9, Canada; 3 Department of Environment and Sustainable Resource Development, Government of Alberta, Edmonton, AB T5K 2M4, Canada; 4 Washington State Department of Ecology, Olympia, WA, 98504-7600, United States of America; 5 Department of Atmospheric and Environmental Sciences, University at Albany, SUNY, Albany, NY, 12222, United States of America; Laval University, CANADA

## Abstract

Air temperatures in the tropical Andes have risen at an accelerated rate relative to the global average over recent decades. However, the effects of climate change on Andean lakes, which are vital to sustaining regional biodiversity and serve as an important water resource to local populations, remain largely unknown. Here, we show that recent climate changes have forced alpine lakes of the equatorial Andes towards new ecological and physical states, in close synchrony to the rapid shrinkage of glaciers regionally. Using dated sediment cores from three lakes in the southern Sierra of Ecuador, we record abrupt increases in the planktonic thalassiosiroid diatom *Discostella stelligera* from trace abundances to dominance within the phytoplankton. This unprecedented shift occurs against the backdrop of rising temperatures, changing atmospheric pressure fields, and declining wind speeds. Ecological restructuring in these lakes is linked to warming and/or enhanced water column stratification. In contrast to seasonally ice-covered Arctic and temperate alpine counterparts, aquatic production has not increased universally with warming, and has even declined in some lakes, possibly because enhanced thermal stability impedes the re-circulation of hypolimnetic nutrients to surface waters. Our results demonstrate that these lakes have already passed important ecological thresholds, with potentially far-reaching consequences for Andean water resources.

## Introduction

**Figure 1:**
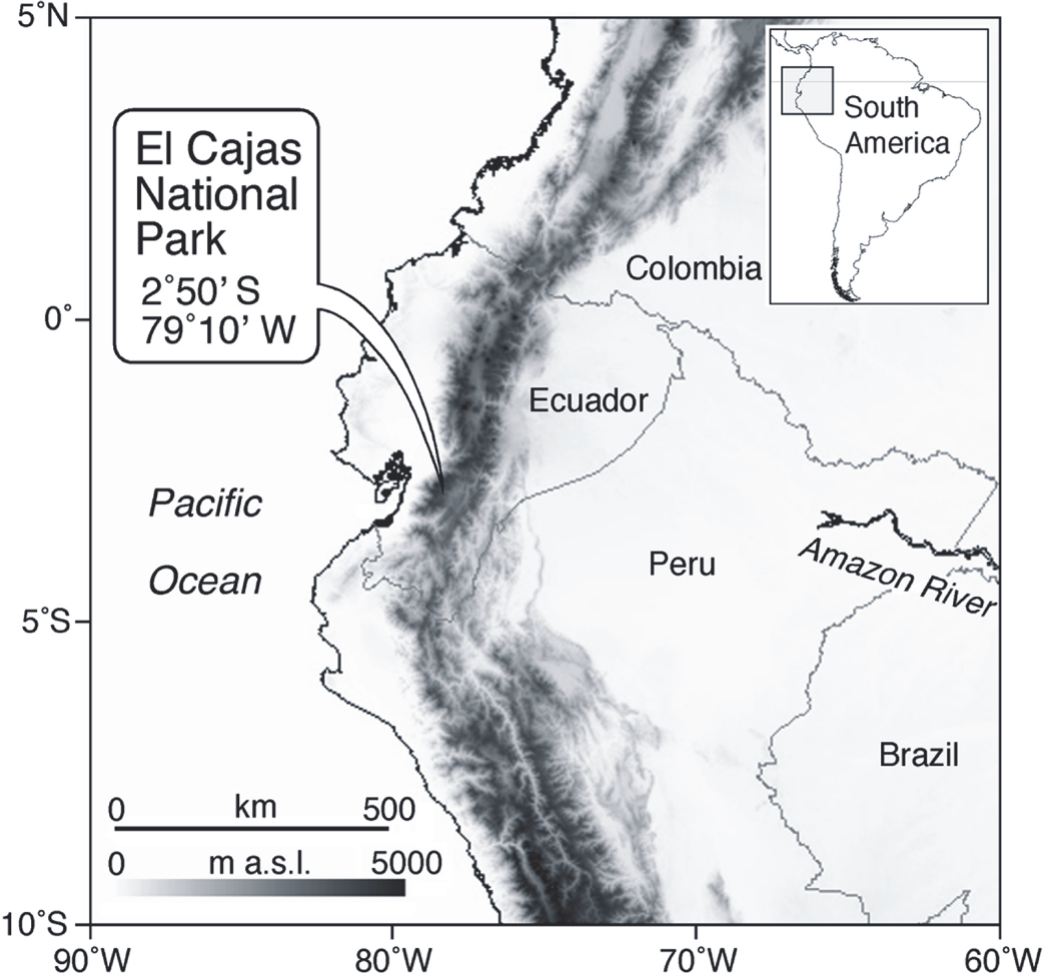
Location of Cajas National Park in the southern Sierra of Ecuador.

Andean societies are amongst the most vulnerable when it comes to the impacts of climate change on freshwater resources. Warming in the Andes is occurring at a rate nearly twice the global average [1, 2], and is already impacting water resources, most notably through the rapid shrinkage of glaciers in recent decades [3–6]. Warming-induced reductions in glacial meltwater have reduced biodiversity and are threatening species extinctions in glacier-fed river systems in Ecuador [7]. However, little is known concerning the consequences of climate change on lakes from this region. Despite their importance for regional water supply, lakes have received relatively little attention within the breadth of ecosystems in the tropical Andes [8]. For example, in Ecuador, the city of Cuenca (population ∼350,000) obtains ∼60% of its drinking water from alpine lakes in nearby Cajas National Park (Fig. 1).

Lake sediments are a widely accessible natural archive of environmental change in the Andes, and paleolimnological research has provided insights into long-term (i.e., millennial-scale) changes in climate-related variables such as El-Niño periodicities [9], South American monsoon history [10], paleohydrology [11], fire dynamics [12], and vegetation history [13, 14]. These studies, among others, have demonstrated that climate in the tropical Andes has been highly dynamic over the Holocene and closely modulated by the Pacific Ocean [15]. However, no studies to date have investigated limnological changes associated with the Holocene-Anthropocene transition [16] in the context of long-term natural variability.

In order to study the impacts of recent warming on equatorial Andean lakes, we recovered sediment cores spanning approximately the last several centuries of limnological change, from three alpine lakes in Cajas National Park (2.83° S, 79.17° W; elevations: 3,100–4,450 m asl) in the Azuay Province of Ecuador (Fig. 1; S1–S3 Figs.). All cores were analyzed for fossil diatom assemblages (Class Bacillariophyceae), single-celled algae considered to be especially sensitive bellwethers of environmental change [17, 18], as well as for concentrations of chlorophyll *a*, a photosynthetic pigment that has been demonstrated to track past changes in whole lake (i.e., ecosystem-scale) production [19].

Cajas National Park contains >200 remote and relatively pristine lakes of glacial origin, isolated from direct human activities in their catchments and thereby obviating the confounding influences of land-uses on nutrient availability. The lakes are accessible only by hiking trails, and fishing is permitted only from shore as boats are prohibited to the general public. There is no development within the catchments, excepting at Llaviacu which has two small buildings (one abandoned) that are rarely used. There are no glaciers within the park, and the lakes are fed mainly by precipitation. The study lakes span an elevation gradient from 3,140 to 3,920 m asl, and have comparable limnological properties, each being circumneutral in pH, dilute, and ultra-oligotrophic (Table A in S1 File).

## Material and Methods

We thank Maria Cecilia Carrasco Espinoza and Juan Carlos Quezada Ledesma at ETAPA-EP Parque Nacional El Cajas for research permits and providing assistance for our field work carried out in July 2011.

Temperature data are from the Cañar meteorological station, the nearest long-term continuous record situated ∼30 km east of Cajas National Park, maintained by the Ecuadorian Meteorological and Hydrological Service (INAMHI). Wind speeds are monthly means from NCEP/NCAR reanalyses [20], at 600 hPa levels, based on a 3°×3° grid average covering Ecuador (0–5° S, 77.5–82.5° W). Data for the Niño 3.4 Region SST anomalies [21], bounded by 120° W-170° W and 5° S- 5° N, were obtained from the Climate Prediction Center, NOAA.

An inflatable dinghy was used to collect both water samples and sediment cores from near the center of each lake. All water and sediment samples were kept cool and dark during the field season in plastic cooler containers. Protocols for bottling, filtering, and methods for chemical analyses followed Environment Canada [22, 23]. Water samples for chemical analyses were collected in pre-cleaned bottles approximatey 30 cm below the surface. Immediately upon returning from the field, water samples were transported to the National Laboratory for Environmental Testing (NLET) in Burlington, ON, Canada for analyses.

Sediment cores were recovered from the deepest portions of each basin using a Universal gravity corer (i.d. = 6.8 cm) and sectioned on-site using a close-interval extruder [24] into 0.5-cm intervals. Core chronology was established using a constant-rate-of-supply (CRS) model applied to excess ^210^Pb inventories (Tables B-D in S2 File), counted on a digital, high-purity germanium γ spectrometer (DSPec, Ortec), following standard procedures [25]. Diatom preparation followed standard protocols for siliceous microfossils [26]; at least 300 diatom valves were identified and enumerated per interval. The percent relative abundance of fossil diatoms for all sediment cores are given in Tables E-G in S3 File.

Total sediment chlorins were analyzed by reflectance spectroscopy using a Model 6500 series Rapid Content Analyzer (FOSS NIRSystems Inc.). In brief, spectroscopic data (400–2500 nm wavelength range) from lyophilized and sieved (125 μm mesh) sediment samples were run through a predictive model based on 35 calibration samples covering a gradient of sediment chlorophyll *a* concentrations, as measured by high performance liquid chromatography. The prediction model was developed using a linear regression between a simple reflectance metric (area under the absorbance peak between 650 and 700 nm) and the summed concentration of total sedimentary chlorophyll *a* and its derivatives. This technique captures both primary and degraded chlorophyll *a* in sediments, so that diagenetic effects are not problematic [27], and has been demonstrated to track climate-related paleoproduction fluctuations on the timescale of the Holocene, as inferred by numerous independent paleoclimate proxies [28]. Spectrally-inferred sediment chlorophyll *a* concentrations for all sediment cores are given in Tables H-J in S4 File.

## Results

**Figure 2:**
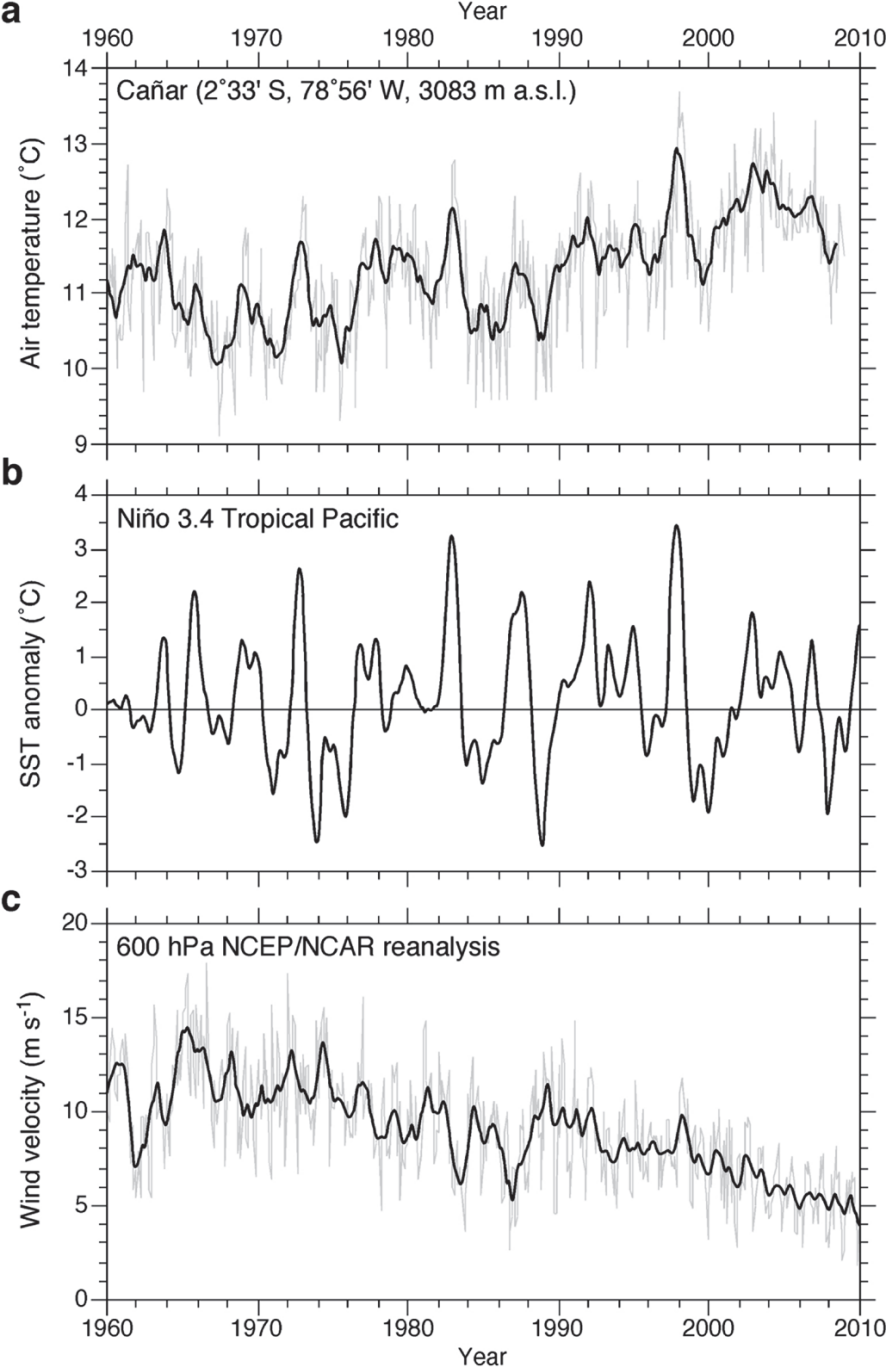
Meteorological records of environmental drivers in the Ecuadorian Andes during the period 1960–2010 AD. **a**, Air temperatures. Monthly averages shown in grey and a nine-month running mean superimposed in black. **b**, Niño 3.4 SST Index. Nine month running mean plotted as departures from the overall mean for the data set (1960–2010). **c**, 600 hPa wind velocity. Monthly averages are shown in grey and a nine-month running mean superimposed in black.

Air temperatures from the Cañar meteorological station near Cajas National Park show a warming trend since the early 1970s with an average increase of 0.29°C per decade, corresponding to a mean annual temperature increase of 1.15°C since that time (Fig. 2A). This increase is comparable to other temperature records across the tropical Andes [2]. Second-order fluctuations in temperature bear a strong relationship to El Niño-driven sea surface temperature (SST) anomalies (Fig. 2B), conferring the direct influence of Pacific Ocean inter-annual variability, superposed on the secular recent warming trend. Concurrent with increasing temperatures, wind velocity has steadily decreased in the Cajas region, dropping by over 40% since 2000 AD relative to the 1960s and 1970s (Fig. 2C).

**Figure 3:**
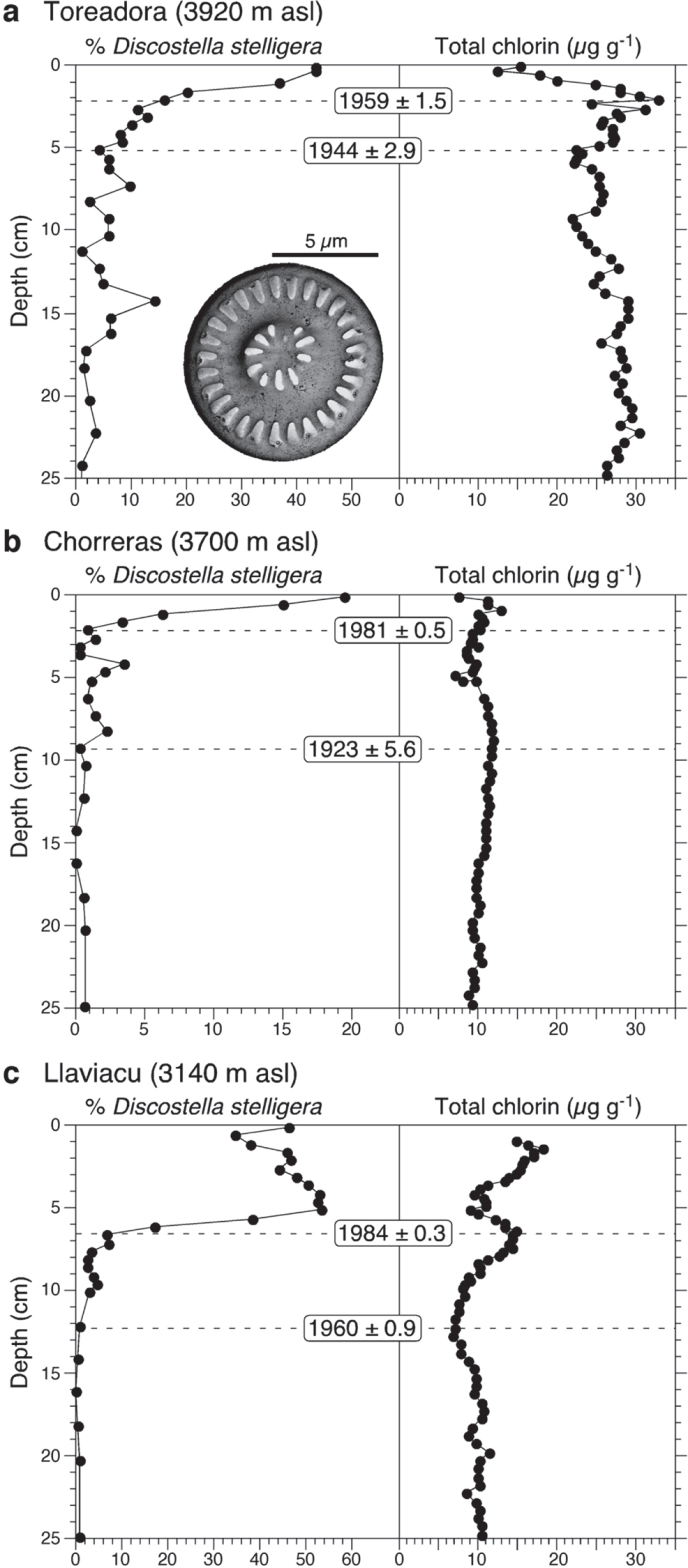
Percent *Discostella stelligera* (relative to total diatoms counted) and total chlorin (chlorophylls and diagenetic products including pheophorbides and pheophytins) concentrations from the investigated sediment cores. **a**, Laguna Toreadora; inset is a photomicrograph of *D. stelligera* under field-emission scanning electron microscopy. **b**, Laguna Chorreras. **c**, Laguna Llaviacu. Horizontal dashed lines indicate key dates for major stratigraphic changes derived from ^210^Pb chronologies, with associated error terms (Tables B-D in S2 File).

**Figure 4:**
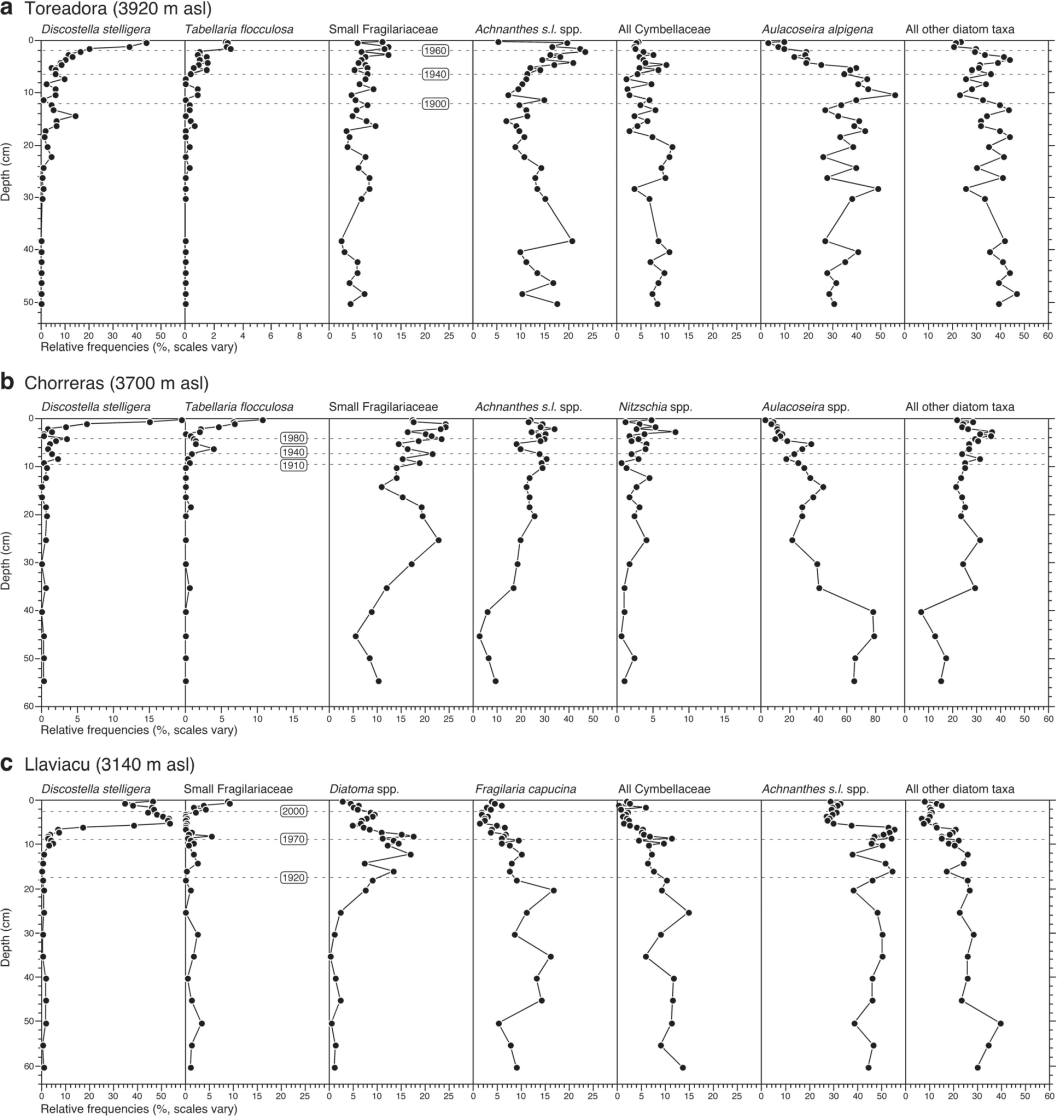
Fossil diatom profiles expressed as percent abundance versus core depth for (a) Toreadora, (b) Chorerras, and (c) Llaviacu. Approximate ^210^Pb dates denoting key stratigraphic changes are inset. (Note: s.l. = *sensu lato*)

High-resolution analyses of diatom microfossil assemblages from dated sediment cores (Tables B-D in S2 File) show that the planktonic species *Discostella stelligera* (basionym: *Cyclotella stelligera*) has increased from trace abundances to become the dominant diatom in all study lakes (Figs. 3, 4). The *Discostella* rise first occurs in the highest elevation lake (Laguna Toreadora, 3,920 m asl) beginning in the early 1960s, and latest in the lowest elevation site (Laguna Llaviuco, 3,140 m asl) during the late 1980s (Fig. 3). In Toreadora and Chorerras, concurrent with the *D. stelligera* rise, there is an increase in *Tabellaria flocculosa* str. IV (Fig. 4A, B). In Llaviacu, the *D. stelligera* rise occurs largely at the expense of benthic diatoms including *Cymbella sensu lato* taxa, *Achnanthes sensu lato* taxa, *Diatoma* taxa and *Fragilaria capucina* (Fig. 4C).

In Toreadora, total sediment chlorin concentrations record consistent declines beginning in the early 1960s and attain unprecedented low levels in the most recent sediments (Fig. 3A). In Chorerras and Llaviacu, sediment chlorin profiles show little-to-no change over the study period (Fig. 3B, C).

## Discussion

The combined effects of increasing temperatures and reduced wind speeds have resulted in marked ecological restructuring in the Cajas study lakes, which are unprecedented within the recent centuries spanned by our sediment archives. All cores record a rapid rise in *D. stelligera* occurring within the last ∼50 years, continuing to the present-day (Fig. 3). Similar increases in the relative abundance of cyclotelloid diatoms have been documented in hundreds of lakes in Europe and North America, beginning in the mid-20th century [29–32]. In these cases, their rise has been attributed to recent climate warming, which has decreased the duration of seasonal ice cover and/or enhanced the stability of water-column thermal stratification in the growing season. Similar species shifts recorded in Cajas National Park indicate that these lakes may be experiencing greater periods of water column stability and thermal stratification as would be expected given rising temperatures and reduced local wind speeds (Fig. 2).

Concurrent with the Discostella rise, sediment cores from Toreadora and Chorerras also record increased in *Tabellaria flocculosa* str. IV (Fig. 4A, B). This taxon is typically classified as planktonic and is commonly documented in the plankton of oligotrophic lakes [33–35]. The increase in *D. stelligera* and *T. flocculosa* occurs at the expense of more heavily silicified tychoplanktonic genera such as *Aulacoseira* that require periodic resuspension by mixing in order to remain in the photic zone [6, 32]. A rapid rise of planktonic diatoms [6, 32], such as the *Discostella* and *Tabellaria* taxa in question, from near zero values to dominance within an assemblage is viewed as a major ecological change because it often reflects a physical restructuring of the water column, which can, in turn, affect a whole suite of lake processes. For example, in tropical lakes, which lack seasonal ice cover, mixing is an essential vector for nutrient supply to the epilimnion [36–38].

Although there are many factors that can affect mixing and stratification, the relationship between elevation and the onset of the *Discostella* and *Tabellaria* rise recorded in the study lakes is consistent with climate models that predict the scaling of temperature increases with altitude [39], and consequently that the highest elevations will be the first to cross climate-driven ecological thresholds. The increase in planktonic taxa, in particular *D. stelligera*, further represents an ecological fingerprint for the onset of the Anthropocene, detectable in many lake sediments of the northern hemisphere [16, 29–32], but hitherto not documented in the tropical Andes. We show here that *Discostella* increases also occur in equatorial alpine lakes where seasonal variation of temperature and solar radiation inputs are minimal, and in the absence of seasonal lake ice cover. Moreover, the limnological changes recorded by the sediment record occur in direct synchrony with climate-related changes recorded elsewhere in the Andes, most notably the rapid demise of glaciers and the up-slope progression of agriculture [3].

Concurrent with diatom assemblage shifts, most northern-hemisphere lakes also record a sharp rise in algal production associated with recent warming [27, 40], driven largely by prolongation of the ice-free growing season. We measured total sediment chlorin (chlorophylls and diagenetic products including pheophorbides and pheophytins) using non-destructive spectroscopic approaches. This serves as a generalized proxy for total algal paleoproduction, in part because it records both primary sedimentary chlorophyll *a* as well as diagenetic products [27, 40]. Trends in whole-lake production inferred from sediment chlorin concentrations do not show increases in aquatic production, and even slight declines in recent decades in the case of Laguna Toreadora (Fig. 3). With climate warming and enhanced thermal stability of the water column, the upwelling of hypolimnetic nutrients to surface waters becomes curtailed, thereby decreasing the sediment signal of total lake production. Although the trend of becoming more oligotrophic with warming has been documented previously in an African rift lake [36, 37], we provide evidence that it may also extend to much smaller tropical alpine lakes.

Our paleoecological data show that warming temperatures and weakening winds have altered the physical and biological structure of tropical Andean lakes in profound ways. Foremost among these changes is the ecological reorganization of phytoplankton communities borne out of sediment diatom assemblages. Attendant changes in nutrient cycling inferred from sediment chlorin concentrations are associated with changes in lake physical mixing processes, such as the strengthening of thermal stratification [37]. Because algae form the base of the food chain in lakes, these changes are harbingers of processes that can reverberate within the trophic structure of lakes, ultimately affecting top predators such as fish, birds, and even humans. Collectively, present and future limnological changes in the Tropical Andes have significant ecological and potentially societal implications that are exacerbated as water availability from glaciers diminishes [4–6]. Given the inevitability of increasing human pressure on lakes regionally, the observation of pronounced ecological restructuring becomes especially sobering.

## Supporting Information

S1 Fig Photograph of Laguna Toreadora. (JPG)

S2 Fig Photograph of Laguna Chorreras. (JPG)

S3 Fig Photograph of Laguna Llaviacu. (JPG)

S1 File Table A. Selected limnological data from the study lakes based on water samples collected from near the center of each site during July 2011.
(XLSX)

S2 File Tables B-D. ^210^Pb radiochemistry and constant rate of supply (CRS) age model for the Laguna Toreadora (Table B), Chorerras (Table C) and Llaviacu (Table D) sediment cores.
(XLSX)

S3 File Tables E-G. Fossil diatom data as percentages for Chorerras (Table E), Llaviacu (Table F), and Toreadora (Table G).
(XLSX)

S4 File Tables H-J. Spectrally-inferred sediment chlorophyll *a* concentrations for Llaviacu (Table H), Toreadora (Table I) and Chorerras (Table J).
(XLSX)
