# Supplementary figures and images for: Climate Change Forces New Ecological States in Tropical Andean Lakes
Source: PLoS One. 2015 Feb 3;10(2):e0115338. doi: 10.1371/journal.pone.0115338 (PMC4315470; doi:10.1371/journal.pone.0115338)

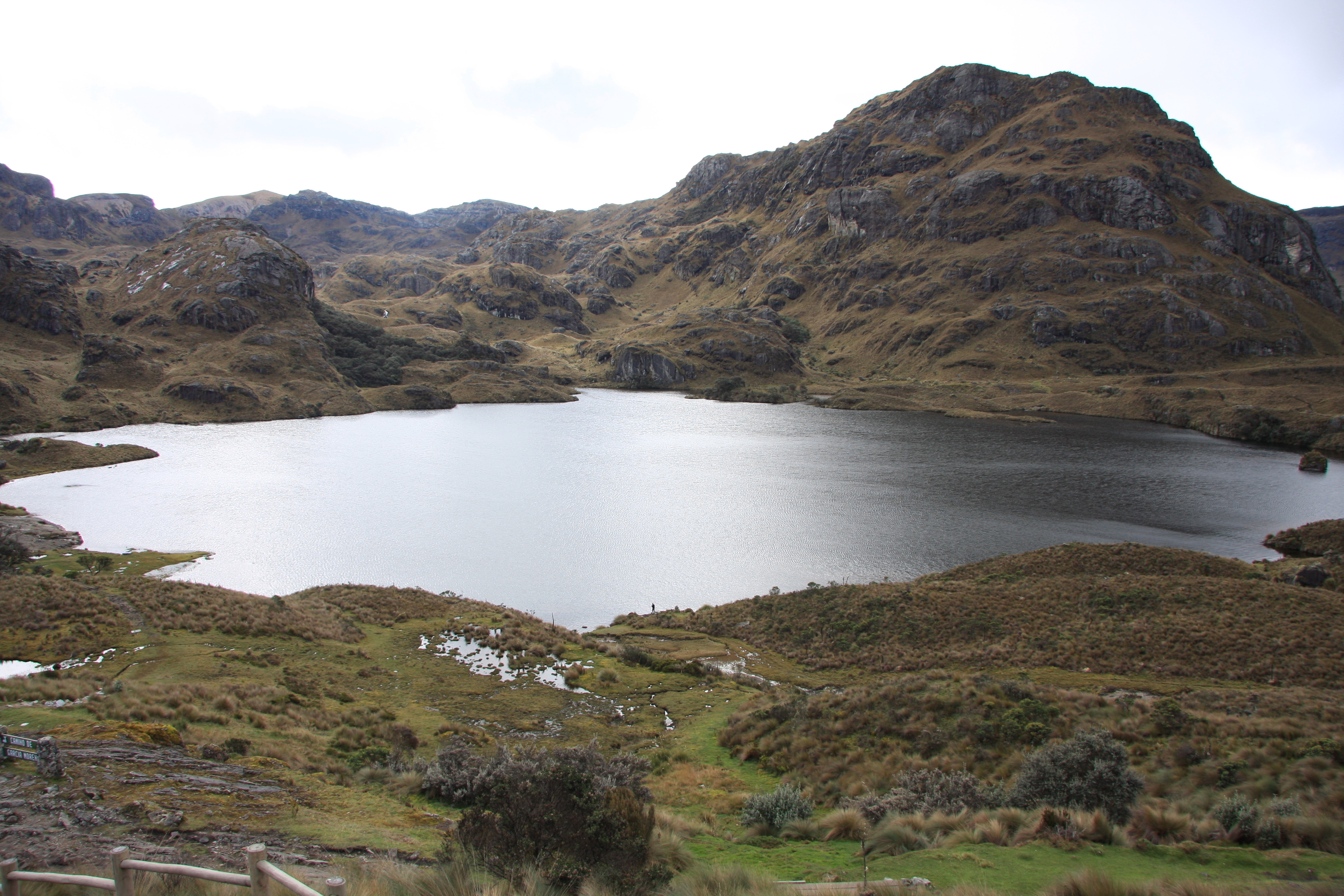

Supplement: S1 Fig — (JPG) [file pone.0115338.s001.jpg]

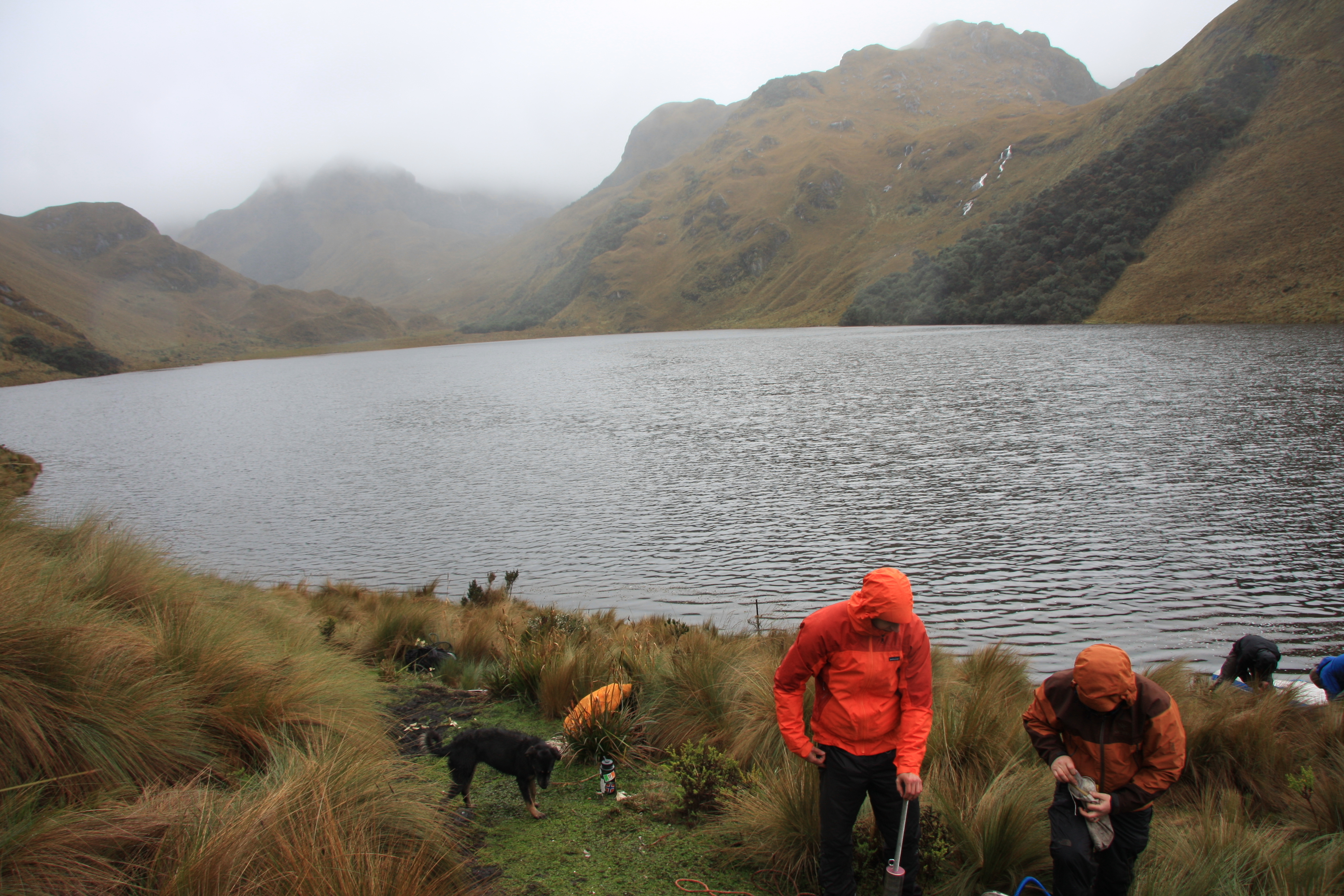

Supplement: S2 Fig — (JPG) [file pone.0115338.s002.jpg]

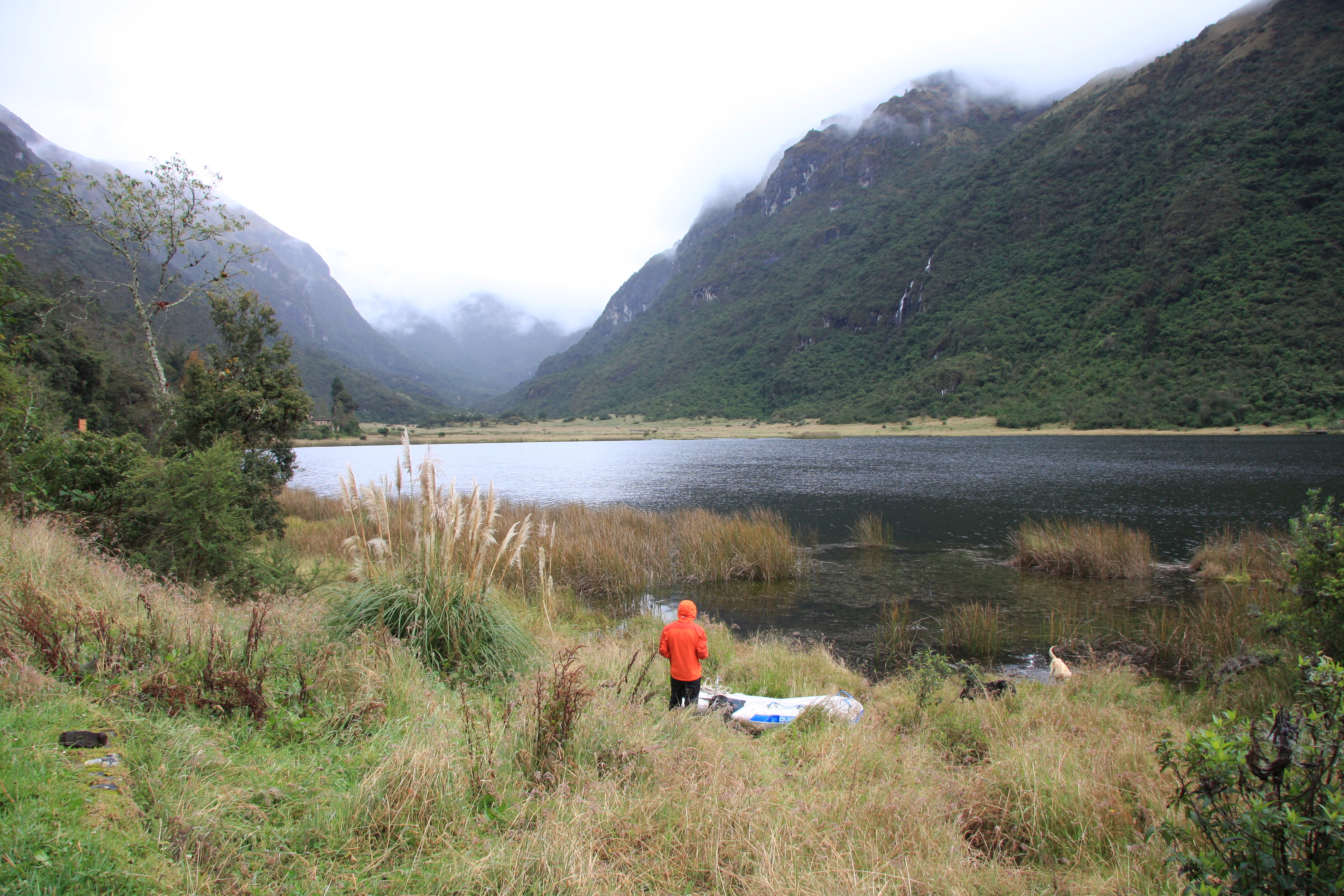

Supplement: S3 Fig — (JPG) [file pone.0115338.s003.jpg]
